# MyD88-Dependent Signaling Influences Fibrosis and Alternative Macrophage Activation during *Staphylococcus aureus* Biofilm Infection

**DOI:** 10.1371/journal.pone.0042476

**Published:** 2012-08-03

**Authors:** Mark L. Hanke, Amanda Angle, Tammy Kielian

**Affiliations:** Department of Pathology and Microbiology, University of Nebraska Medical Center, Omaha, Nebraska, United States of America; Indian Institute of Science, India

## Abstract

Bacterial biofilms represent a significant therapeutic challenge based on their ability to evade host immune and antibiotic-mediated clearance. Recent studies have implicated IL-1β in biofilm containment, whereas Toll-like receptors (TLRs) had no effect. This is intriguing, since both the IL-1 receptor (IL-1R) and most TLRs impinge on MyD88-dependent signaling pathways, yet the role of this key adaptor in modulating the host response to biofilm growth is unknown. Therefore, we examined the course of *S. aureus* catheter-associated biofilm infection in MyD88 knockout (KO) mice. MyD88 KO animals displayed significantly increased bacterial burdens on catheters and surrounding tissues during early infection, which coincided with enhanced dissemination to the heart and kidney compared to wild type (WT) mice. The expression of several proinflammatory mediators, including IL-6, IFN-γ, and CXCL1 was significantly reduced in MyD88 KO mice, primarily at the later stages of infection. Interestingly, immunofluorescence staining of biofilm-infected tissues revealed increased fibrosis in MyD88 KO mice concomitant with enhanced recruitment of alternatively activated M2 macrophages. Taken in the context of previous studies with IL-1β, TLR2, and TLR9 KO mice, the current report reveals that MyD88 signaling is a major effector pathway regulating fibrosis and macrophage polarization during biofilm formation. Together these findings represent a novel example of the divergence between TLR and MyD88 action in the context of *S. aureus* biofilm infection.

## Introduction

*Staphylococcus aureus* (*S. aureus*) represents a major cause of both health care- and community-associated infections and with the increased prevalence of methicillin-resistant *S. aureus* (MRSA), this pathogen has become an even greater therapeutic challenge [1]. Currently, MRSA accounts for greater than 50% of all *S. aureus* isolates causing nosocomial infections that can manifest as sepsis, endocarditis, skin and soft tissue infections, and osteomyelitis [2]. In addition, infection risk is increased by the presence of foreign materials, and *S. aureus* is a frequent etiological agent of biofilm infections on indwelling devices and artificial joints that are especially problematic because of their persistence and recalcitrance to conventional antibiotic therapy [3], [4], [5].

*S. aureus* biofilms are complex bacterial communities encased in a matrix composed primarily of polysaccharides, extracellular DNA (eDNA), and proteins [6], [7], [8], [9]. Many of these motifs are recognized by the innate immune system via the Toll-like receptor (TLR) family of pattern recognition receptors, which induces the secretion of numerous proinflammatory mediators that recruit and activate immune cell populations to sites of infection [10], [11], [12]. Although ligands for both TLR2 and TLR9 are present within *S. aureus* biofilms [5], [6], [9] studies from our laboratory and others have demonstrated that neither receptor impacts biofilm growth *in vivo* [13], [14]. In contrast, recent evidence indicates that IL-1β plays a protective role during *S. aureus* biofilm formation in a post-surgical joint infection model [14]. This is intriguing, since both TLRs and IL-1β converge on MyD88-dependent signaling pathways, which prompted our investigation into the functional impact of MyD88 during the course of *S. aureus* biofilm infection.

Here we demonstrate that MyD88 influences the course of *S. aureus* catheter-associated biofilm infection. Specifically, bacterial burdens were significantly increased on indwelling catheters and surrounding tissues of MyD88 knockout (KO) compared to wild type (WT) mice during early stages of infection, which coincided with enhanced dissemination to the heart and kidney. The expression of several proinflammatory mediators was decreased in biofilm-infected tissues of MyD88 KO mice and immunofluorescence staining revealed an increased fibrotic response in MyD88 KO animals concomitant with enhanced recruitment of alternatively activated M2 macrophages. These studies advance our understanding of the hierarchy of MyD88-dependent pathways and, in the context of earlier work with TLR and IL-1β KO animals, suggest that MyD88 plays a dual role during biofilm infection. First, MyD88-dependent signals are responsible for early biofilm containment as typified by increased titers and infection dissemination in MyD88 KO animals. In addition, MyD88 normally induces M1 macrophage polarization, which is revealed by exaggerated M2 macrophage infiltrates and extensive fibrosis following MyD88 loss. Collectively, these findings indicate that the lack of MyD88 augments macrophage polarization towards an anti-inflammatory/pro-fibrotic M2 phenotype, which may impede bacterial clearance and contributes to biofilm persistence *in vivo*, in part, by enhancing the fibrotic encapsulation of biofilm infections.

**Figure 1:**
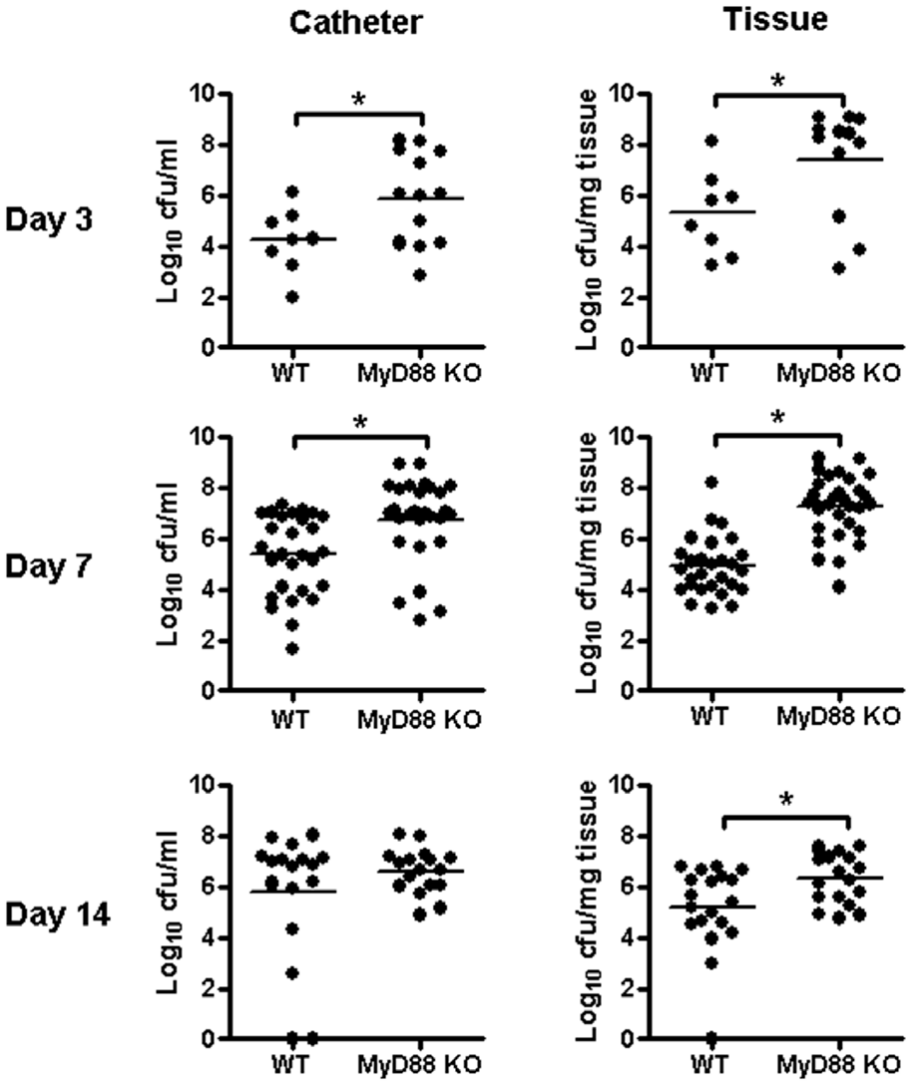
MyD88 regulates acute *S. aureus* biofilm development. Biofilm infections were established in MyD88 knockout (KO) and wild type (WT) mice following the inoculation of 10^3^ CFU of USA300 LAC::*lux* into the lumen of subcutaneous implanted catheters. Animals were sacrificed at the indicated days following *S. aureus* infection, whereupon catheters and surrounding host tissues were removed to quantitate bacterial burdens. Results are expressed as the number of CFU per ml of fluid used for sonication (for catheters) or CFU per mg host tissue to correct for differences in tissue sampling size. Results are presented from individual animals in each group combined from a total of 3 independent experiments with bars representing the mean of each group. Significant differences in bacterial burdens between MyD88 KO and WT mice are denoted by asterisks (*p<0.05).

## Results

### MyD88-dependent Signaling Influences Early *S. aureus* Biofilm Growth

**Figure 2:**
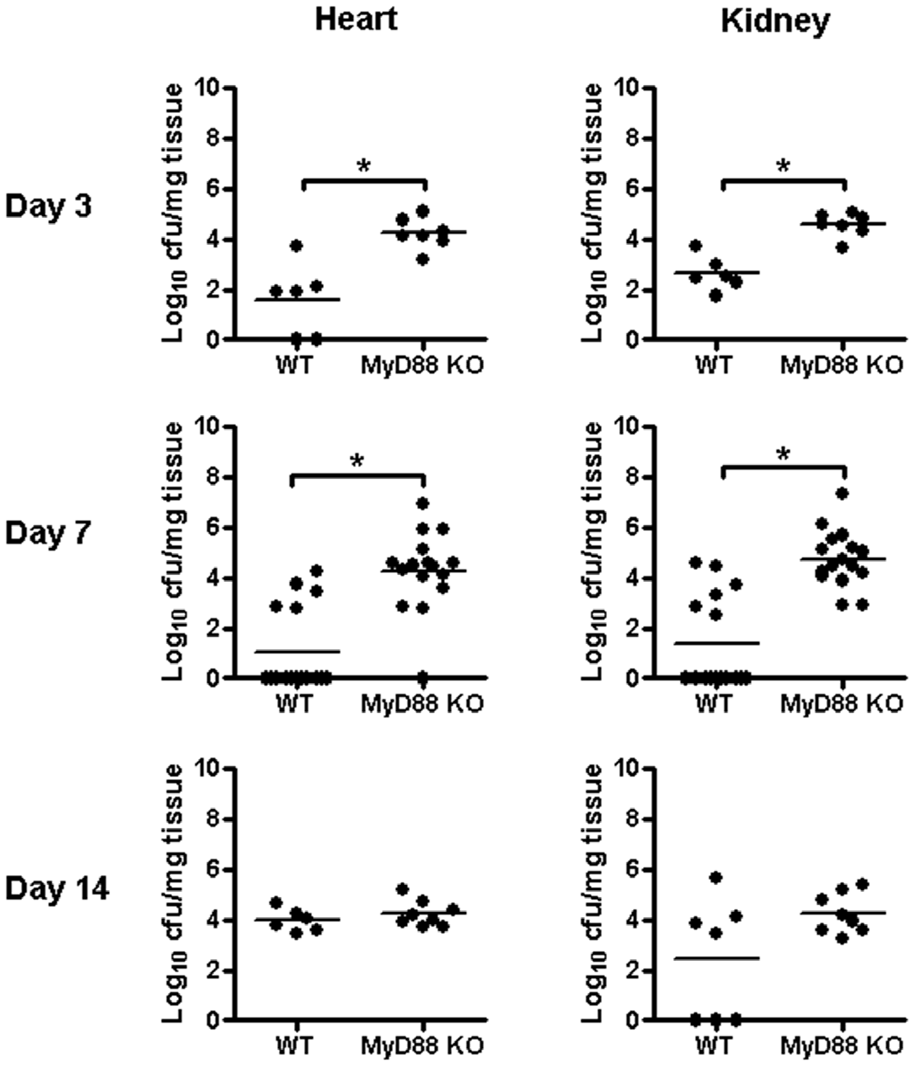
MyD88 loss leads to early impairments in *S. aureus* containment at the site of biofilm infection. Biofilm infections were established in MyD88 knockout (KO) and wild type (WT) mice following the inoculation of 10^3^ CFU of USA300 LAC::*lux* into the lumen of subcutaneous implanted catheters. Animals were sacrificed at the indicated days following *S. aureus* biofilm infection, whereupon the heart and kidneys were removed to quantitate bacterial burdens with results expressed as CFU per mg tissue. Results are presented from individual animals in each group combined from a total of at least 2 independent experiments with bars representing the mean of each group. Significant differences in bacterial burdens between MyD88 KO and WT mice are denoted by asterisks (*p<0.05).

Prior studies examining innate immunity to *S. aureus* biofilms have revealed an intriguing dichotomy. Namely, biofilms evade TLR2- and TLR9-mediated recognition, whereas IL-1β-dependent pathways regulate *S. aureus* biofilm growth [13], [14]. Since these receptors all converge to utilize MyD88-dependent signals, it remained unclear whether this adaptor was critical for anti-biofilm activity, which was the objective of the current study. To address this issue, we evaluated the course of *S. aureus* biofilm growth and dissemination in MyD88 KO mice. Bacterial burdens on both infected catheters and surrounding tissues were significantly elevated in MyD88 KO compared to WT animals at days 3 and 7 post-infection and extended out to day 14 in biofilm-infected tissues of the former (Fig. 1). In contrast, no significant differences in bacterial burdens were observed between the groups at day 21 after infection (data not shown). These results demonstrate that MyD88 is critical during early infection for controlling both biofilm density and bacteria that have become detached from the biofilm, the latter representing a critical step for infection dissemination. Indeed, systemic colonization of the heart and kidney from the primary site of biofilm infection was significantly enhanced in MyD88 KO mice at days 3 and 7 (Fig. 2), revealing a role for MyD88-dependent signals in early biofilm dissemination. The degree of *S. aureus* spread was similar between MyD88 KO and WT animals at days 14 and 21 (Fig. 2 and data not shown), again highlighting the importance of MyD88 action during acute biofilm infection.

**Figure 3:**
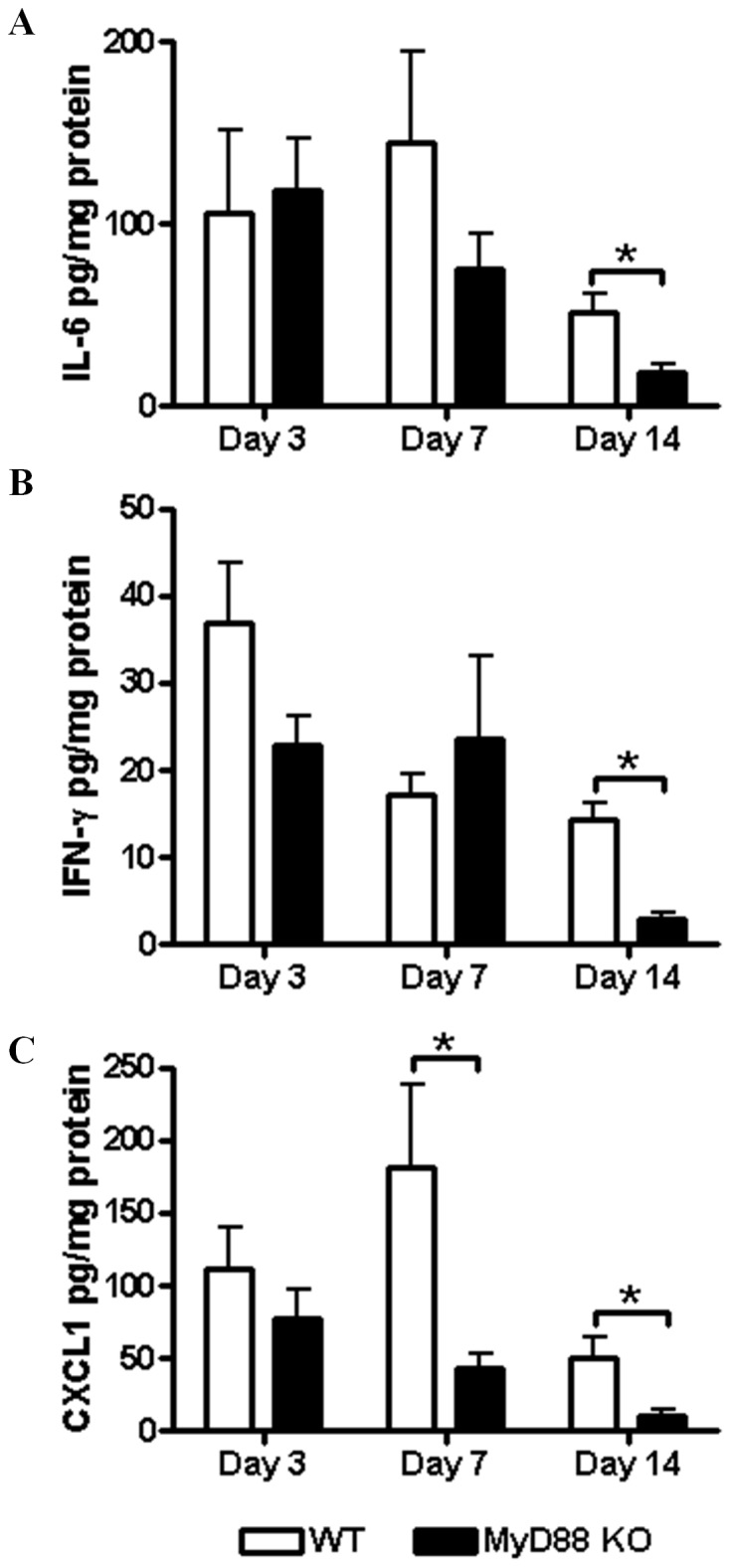
MyD88-dependent signals are important for cytokine and chemokine production during later stages of *S. aureus* biofilm infection. Tissues surrounding *S. aureus* catheter-associated biofilm infections from MyD88 KO and WT mice were collected at the indicated time points post-infection to quantitate differences in IL-6 (A), IFN-γ (B), and CXCL1 (C) expression by MILLIPLEX analysis. Results are normalized to the amount of total protein recovered to correct for differences in tissue sampling size and represent the mean values from individual animals combined from two independent experiments (n = 6−10 per group). Significant differences between MyD88 KO and WT mice are denoted by asterisks (*p<0.05).

Interestingly, although bacterial burdens were altered in MyD88 KO animals at acute time points (Fig. 1), cytokine (IL-6, and IFN-γ) and chemokine (CXCL1) expression was only significantly reduced in biofilm-infected tissues at later intervals (Fig. 3). Therefore, MyD88-dependent signal(s) appear responsible, in part, for IFN-γ, IL-6, and CXCL1 expression during late infection, although the mechanism(s) involved for targeting these factors remain to be identified. Taken in the context of previous studies with TLR2 and TLR9 KO animals where cytokine/chemokine expression was not affected, the current report advances our understanding of MyD88-dependent cascades in dictating inflammatory mediator release in response to *S. aureus* biofilm infections that do not involve TLRs.

**Figure 4:**
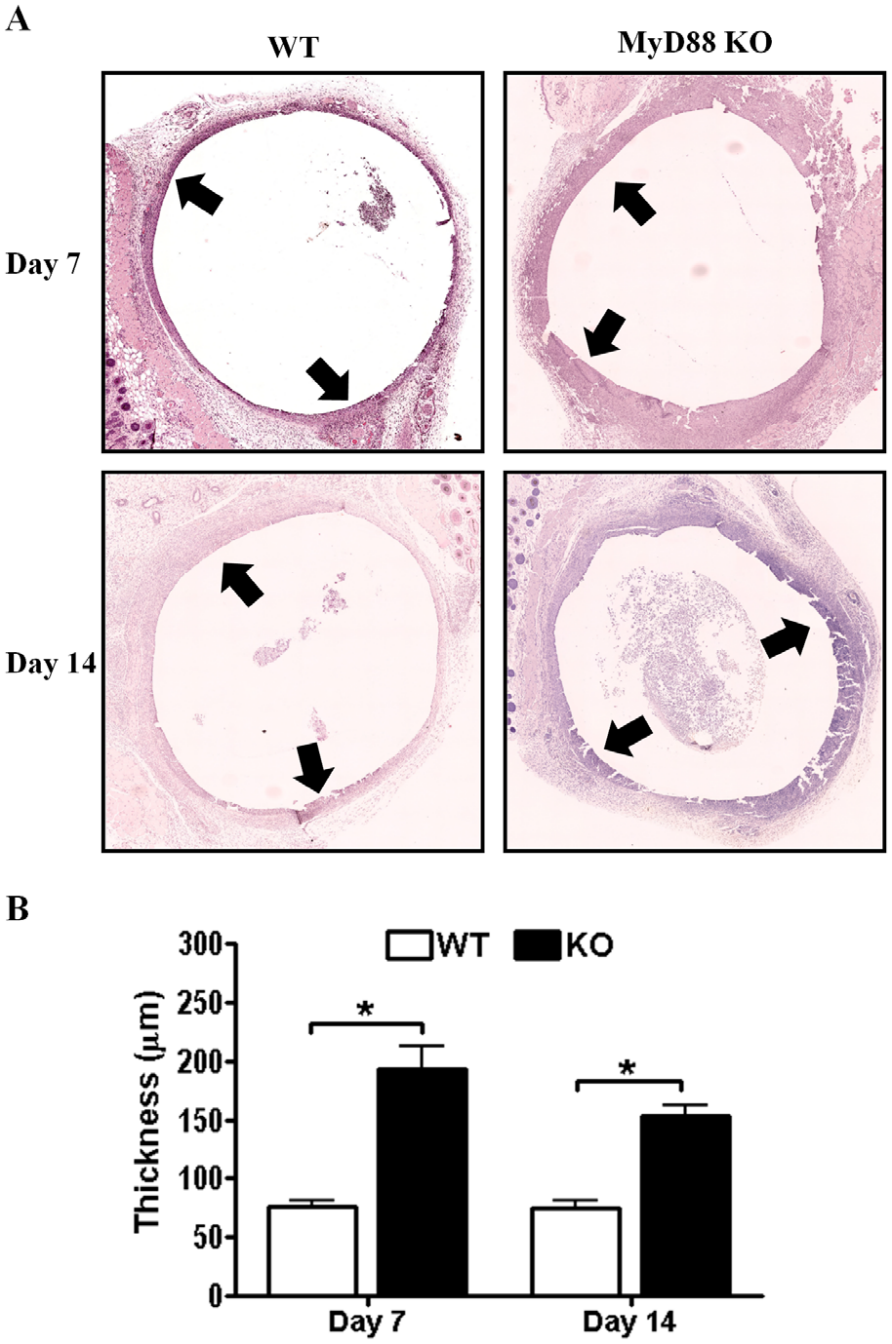
MyD88-dependent signals influence the host tissue response to *S. aureus* biofilms. Biofilm-infected tissues were recovered from MyD88 knockout (KO) and wild type (WT) mice at the indicated time points following infection, whereupon sections were processed by hematoxylin and eosin (H&E) staining to demonstrate changes in tissue architecture (A). The deposition of host material surrounding infected catheters is denoted by arrows. (B) Quantitation of the fibrotic thickness surrounding *S. aureus* biofilms of MyD88 KO and WT mice. Results represent measurements taken from at least seven individual animals per group for each time point where significant differences are indicated with asterisks (*p<0.05).

**Figure 5:**
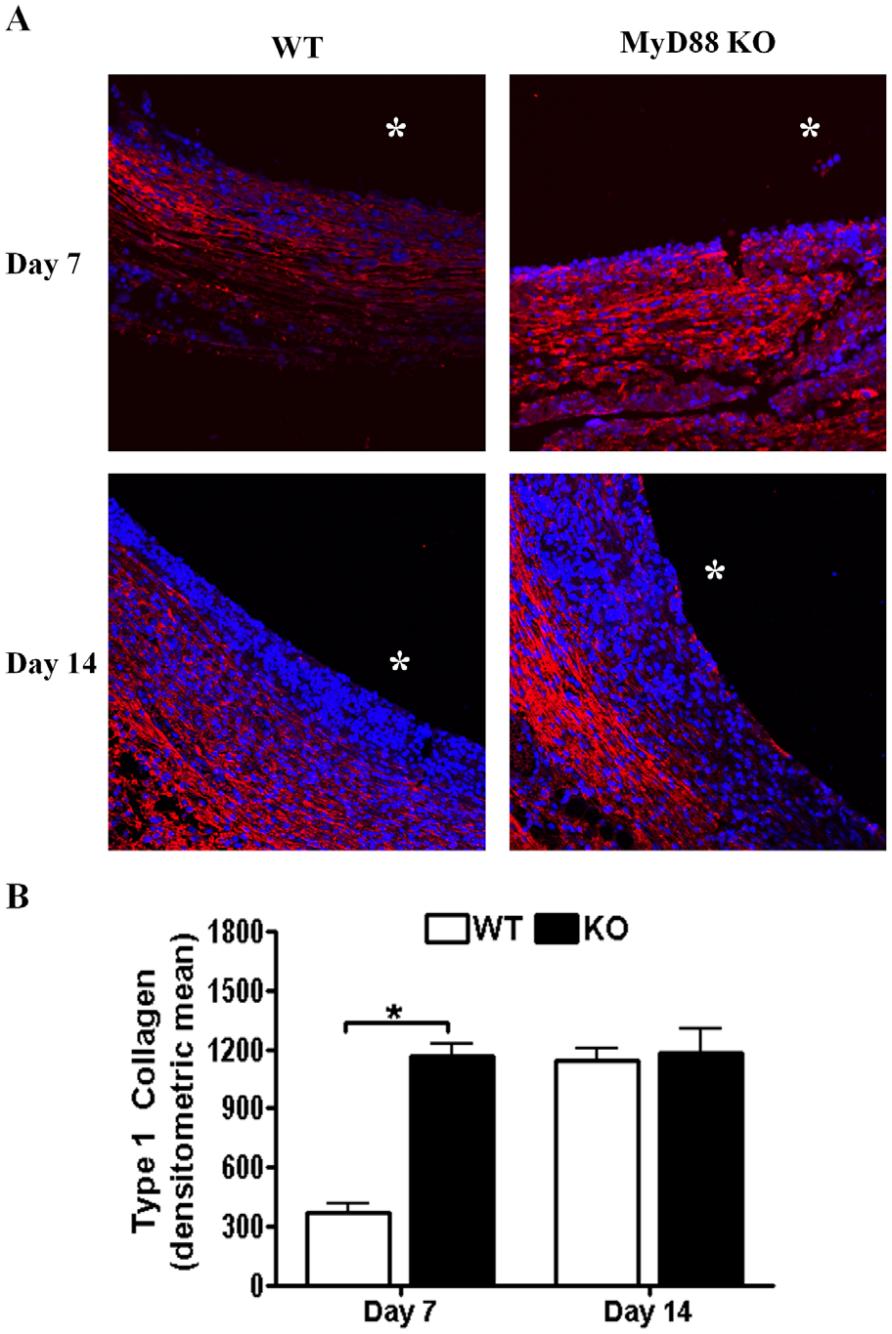
Type I collagen deposition surrounding *S. aureus* biofilms is enhanced following MyD88 loss. Biofilm-infected tissues were recovered from MyD88 knockout (KO) and wild type (WT) mice at the indicated time points following infection, whereupon sections were processed by immunofluorescence staining for type I collagen (red; A). Tissues were stained with DAPI (blue) to demarcate nuclei and asterisks denote the original location of infected catheters that were non-adherent to glass slides. (B) Quantitation of type I collagen fluorescence surrounding *S. aureus* biofilms of MyD88 KO and WT mice. Results are representative of tissues collected from five individual animals per group for each time point where significant differences are indicated with an asterisk (*p<0.05).

### MyD88-dependent Signals Influence the Host Fibrotic Response to *S. aureus* Biofilms

A recent report demonstrated enhanced fibrosis in the lungs of IL-1RI KO animals during *C. pneumoniae* infection. Since this receptor requires MyD88 for signaling and biofilm infections in humans as well as our mouse model are associated with a fibrotic response, tissue morphology surrounding infected catheters of MyD88 KO and WT mice was examined by H&E staining. MyD88 KO mice exhibited increased cellularity at the biofilm-host tissue interface that was significantly increased in thickness compared to WT animals (Fig. 4). Biofilm-infected catheters were encapsulated in a fibrous matrix composed primarily of type I collagen and fibronectin at days 7 and 14 following infection (Fig. 5 and data not shown). The degree of type I collagen deposition was significantly increased in MyD88 KO mice at day 7 post-infection (Fig. 5); however, type I collagen levels were similar between WT and MyD88 KO mice at day 14, again indicating an early effect of MyD88 loss. These results reveal a role for MyD88 signaling in influencing the extent of extracellular matrix (ECM) deposition and fibrosis during early *S. aureus* biofilm infection.

**Figure 6:**
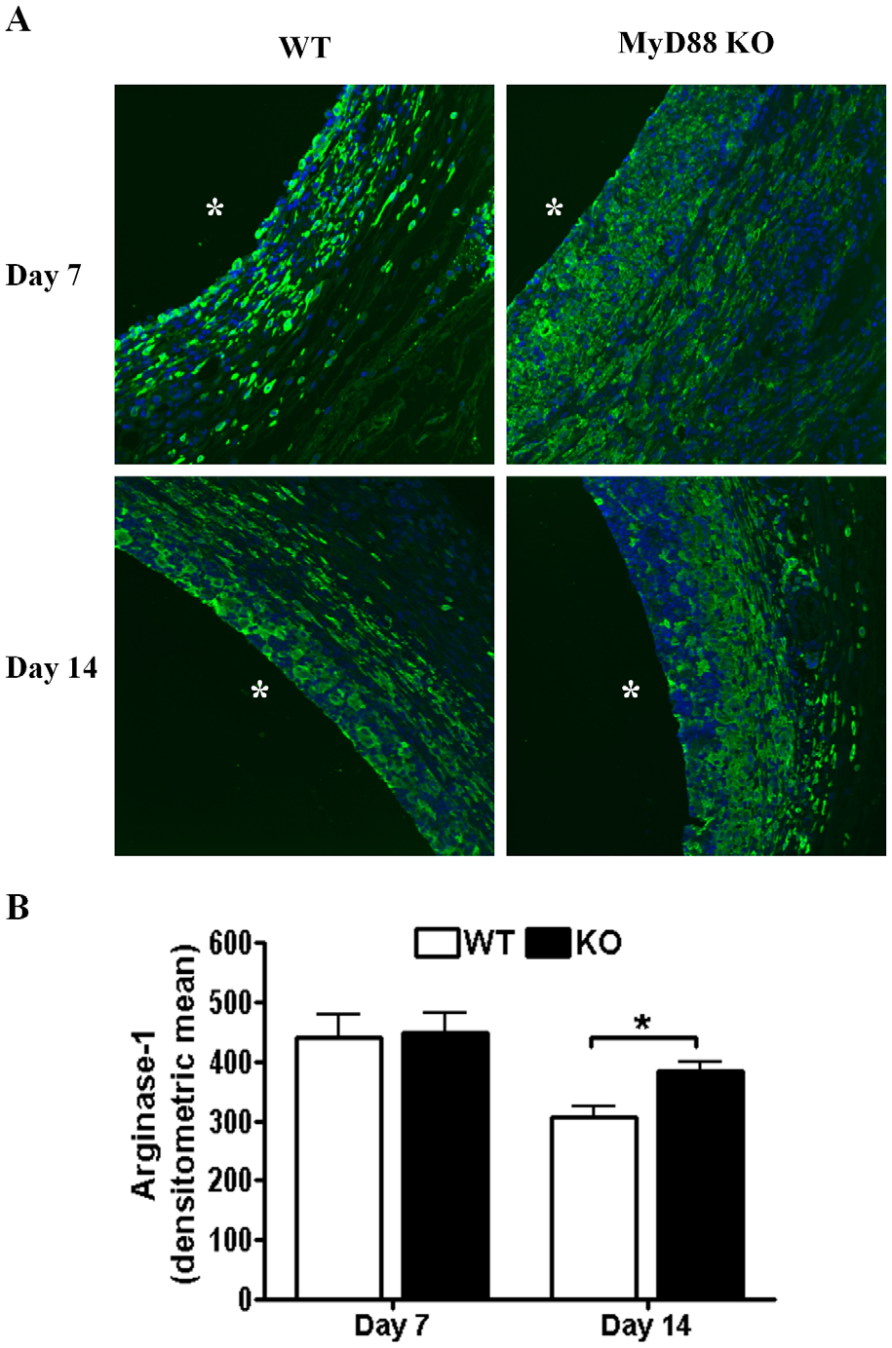
Arginase-1 expression is increased in MyD88 KO mice during *S. aureus* biofilm infection. Biofilm-infected tissues were recovered from MyD88 knockout (KO) and wild type (WT) mice at the indicated time points following infection, whereupon sections were processed by immunofluorescence staining for arginase-1 (green; A). Tissues were stained with DAPI (blue) to demarcate nuclei and asterisks denote the original location of infected catheters that were non-adherent to glass slides. (B) Quantitation of arginase-1 fluorescence surrounding *S. aureus* biofilms of MyD88 KO and WT mice. Results are representative of tissues collected from five individual animals per group for each time point where significant differences are indicated with an asterisk (*p<0.05).

**Figure 7:**
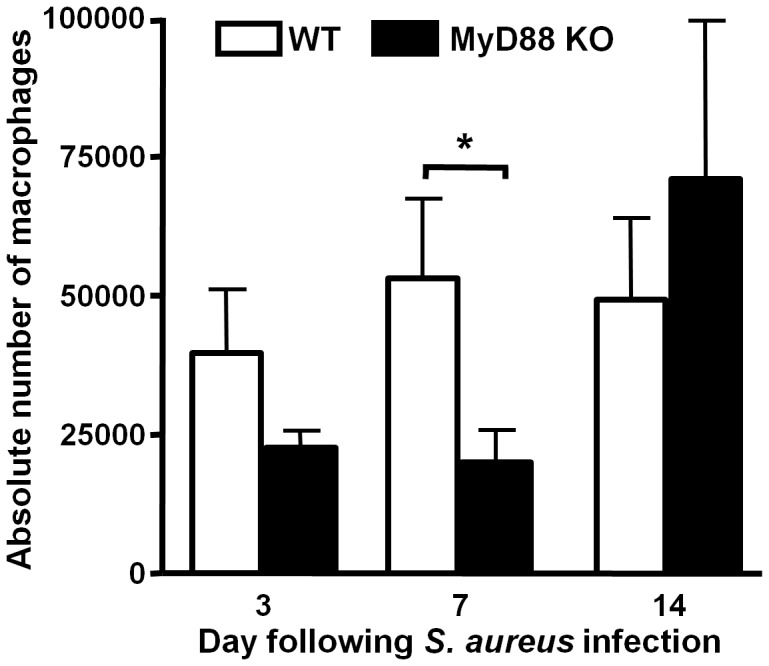
Macrophage recruitment into *S. aureus* biofilms is attenuated in MyD88 KO mice during early infection. Biofilms were established in MyD88 knockout (KO) and wild type (WT) mice following the inoculation of 10^3^ CFU of USA300 LAC::*lux* into the lumen of subcutaneous implanted catheters. Animals were sacrificed at the indicated time points following *S. aureus* infection, whereupon tissues surrounding infected catheters were collected to quantitate macrophage infiltrates by FACS. Results are expressed as the absolute number of F4/80^+^ macrophages after normalization to adjust for the recovery of different cell numbers from MyD88 KO and WT mice and represent the mean ± SEM of three independent experiments. Significant differences in macrophage infiltrates are denoted by an asterisk (*p<0.05).

### MyD88 Loss Results in Excessive Macrophage Polarization Towards an Alternatively Activated M2 Phenotype

**Figure 8:**
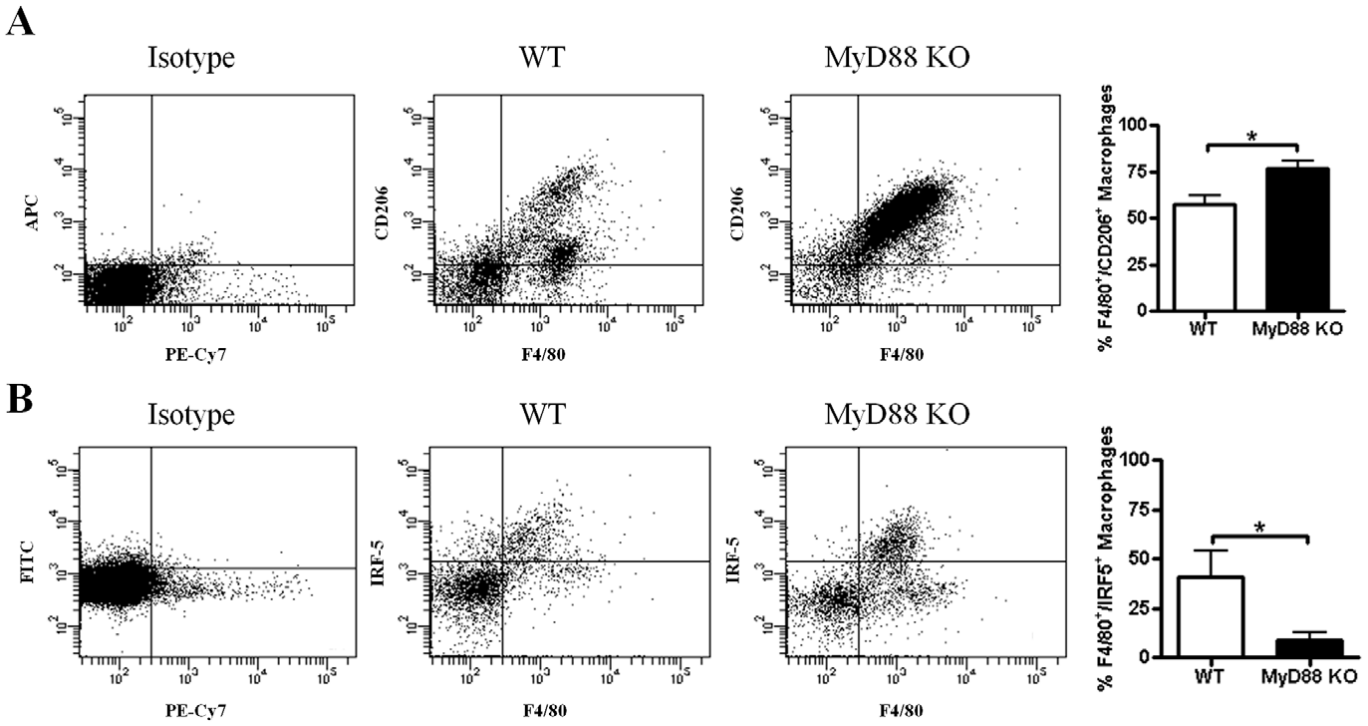
MyD88 loss during *S. aureus* biofilm infection augments alternatively activated M2 macrophage accumulation. Biofilm infections were established in MyD88 knockout (KO) and wild type (WT) mice following the inoculation of 10^3^ CFU of USA300 LAC::*lux* into the lumen of subcutaneous implanted catheters. Animals were sacrificed at day 7 following *S. aureus* infection, whereupon tissues surrounding infected catheters were collected to quantitate M2 (A) and M1 (B) macrophage infiltrates by FACS on the basis of CD206 and IRF-5 staining, respectively. Results are expressed as the percent macrophages (F4/80^+^) that were also positive for CD206 or IRF-5 after correction for isotype control staining and represent the mean ± SEM of three independent experiments. Significant differences between MyD88 KO and WT infiltrates are denoted by asterisks (*p<0.05).

Alternatively activated M2 macrophages participate in fibrotic responses and wound healing, in part, by their expression of arginase-1. Arginase-1 is involved in the collagen biosynthetic pathway for ECM synthesis and fibrosis [15], [16], [17] and skews macrophages away from microbicidal activity, since it competes with the antimicrobial effector iNOS for the shared substrate arginine [18]. Therefore, we next examined whether MyD88 loss altered arginase-1 expression as a potential mechanism to explain the observed increases in ECM deposition and bacterial burdens associated with biofilm infections in MyD88 KO animals. Arginase-1 expression was significantly increased in MyD88 KO mice at day 14 following infection but not earlier (Fig. 6). To further investigate whether MyD88 loss augmented M2 macrophage polarization, we first determined the frequency of macrophage infiltrates by FACS. Biofilm infections in MyD88 KO animals were associated with a significant reduction in the number of infiltrating macrophages at day 7 post-infection (Fig. 7), which were biased towards an anti-inflammatory/pro-fibrotic phenotype as revealed by a significant increase in CD206^+^ expression concomitant with decreased IRF-5^+^ levels (Fig. 8A and B, respectively). These findings demonstrate that MyD88-dependent signals regulate the balance of M1–M2 macrophage polarization during *S. aureus* biofilm infections and that loss of this adaptor activity leads to the preferential accumulation of M2 macrophages, which corroborates the enhanced fibrosis observed in biofilm infected tissues of MyD88 KO animals (Fig. 4).

**Figure 9:**
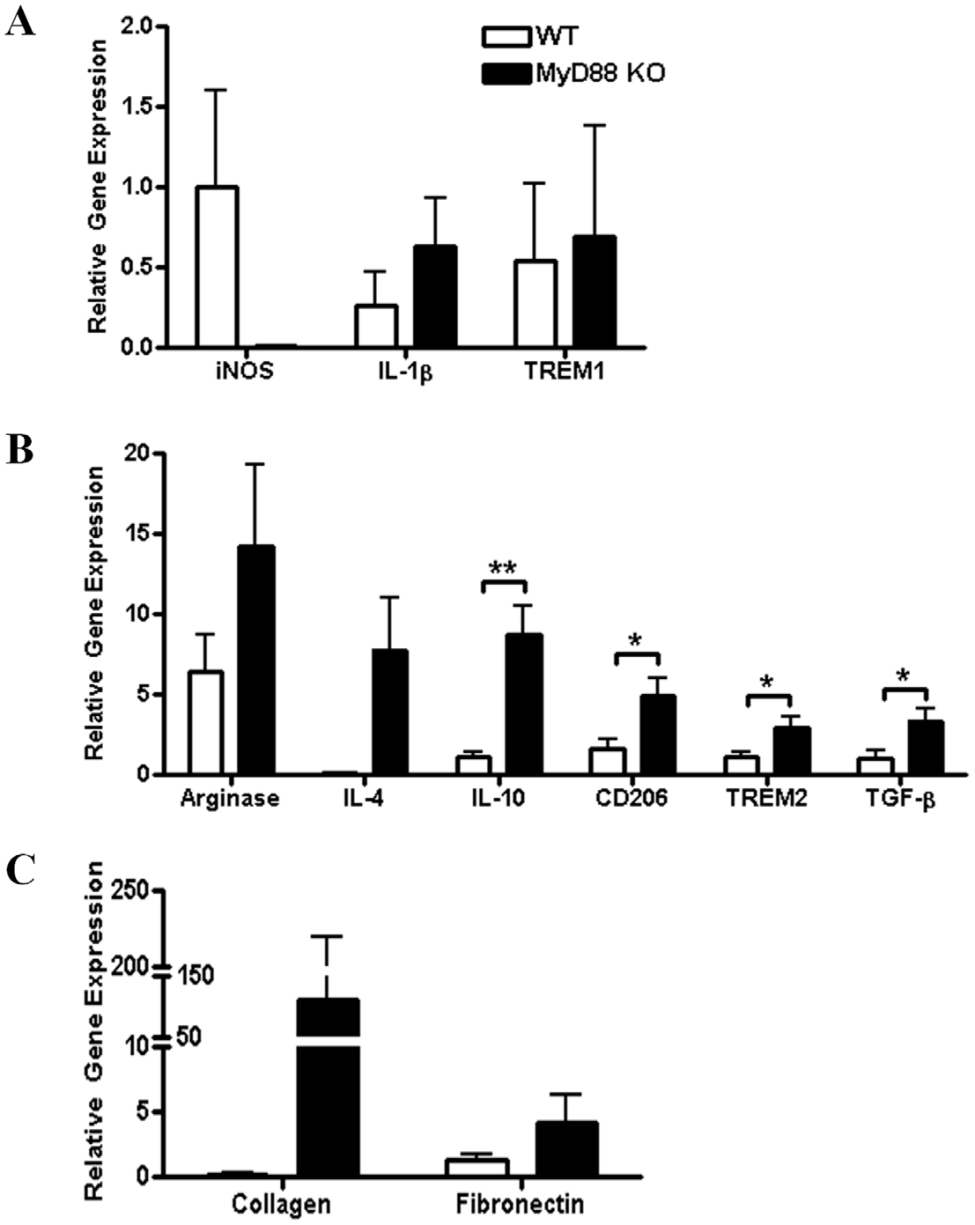
Loss of MyD88-dependent signaling augments the expression of genes associated with alternatively activated M2 macrophages following *S. aureus* biofilm exposure. Bone marrow-derived macrophages from MyD88 KO or WT mice were co-cultured with *S. aureus* biofilms *in vitro* for 2 h, whereupon viable macrophages were purified by FACS and RNA immediately isolated for qRT-PCR analysis. The expression levels of M1 (A), M2 (B), and extracellular matrix (C) genes in macrophages exposed to *S. aureus* biofilms were calculated after normalizing signals against GAPDH and are presented as fold-change relative to unstimulated macrophages. Results represent the mean ± SEM of at least two independent experiments (*p<0.05; **p<0.001).

As an independent means to confirm that MyD88 loss favors M2 macrophage polarization, we performed *in vitro* co-culture studies using macrophages from MyD88 KO or WT mice with *S. aureus* biofilms. MyD88 KO macrophages co-cultured with biofilms demonstrated increased expression of numerous M2-associated genes, including IL-10, CD206, TREM-2, and TGF-β (Fig. 9B). Expression of the ECM molecules type I collagen and fibronectin was higher in MyD88 KO macrophages, although these differences did not reach statistical significance (Fig. 9C), whereas no significant alterations in M1 genes were observed (Fig. 9A). Together these findings reveal that MyD88 signaling regulates fibrosis and macrophage polarization during biofilm formation, which likely influences biofilm persistence. The identity of these MyD88-dependent pathways remains to be defined; however, it is clear from previous work that TLR2 and TLR9 are not involved and as alternative TLRs are not triggered by *S. aureus*, this leaves the possibility of IL-1RI, IL-18R, and IL-33R to be examined in future studies.

## Discussion

Biofilm infections are associated with a high morbidity, with surgical debridement or implant removal in conjunction with long-term antibiotic regimens representing the most successful therapies [19], [20], [21]. Limited information is currently available regarding innate immune responses to *S. aureus* biofilms, which may prove critical for developing new therapeutic modalities for infection. To our knowledge, this study is the first to report the involvement of MyD88-dependent signals in orchestrating innate immune responses to *S. aureus* biofilm infections. This was an important issue to address in the context of recent evidence demonstrating that IL-1β, but not TLR2 or TLR9, affected *S. aureus* biofilm development [13], [14]. Because these molecules all converge on MyD88 for signal transduction, addressing the importance of MyD88 during biofilm growth may elucidate key pathways for novel biofilm therapeutics. In particular, bacterial burdens on catheters and surrounding tissues were significantly elevated in MyD88 KO mice during acute infection, which correlated with a failure in infection containment, as evidenced by increased dissemination to the kidney and heart at days 3 and 7 post-infection. Similar increases in bacterial burdens on infected pins and surrounding tissues in MyD88 KO mice were also observed using an orthopedic model of *S. aureus* biofilm infection (data not shown). Interestingly, histological analysis revealed an exaggerated host fibrotic response surrounding infected catheters of MyD88 KO animals, which agreed with a bias towards alternatively activated M2 macrophages that are known to promote fibrosis. Together, these results indicate that signals emanating from MyD88 are involved in orchestrating several aspects of the host response to *S. aureus* biofilm infection, including bacterial containment, fibrosis, and M2 macrophage polarization. In the absence of MyD88, these responses become dysregulated leading to increased bacterial burdens and infection dissemination during acute infection, although it remains to be determined whether these actions are direct or indirect. Taken in the context of previous studies, the current report allows us to predict key MyD88-dependent pathways that regulate *S. aureus* biofilm development. For example, TLR2 and TLR9 have no influence on biofilm progression, yet MyD88 does. Based on the known ligand specificity of TLRs, the only remaining TLRs that could affect *S. aureus* biofilm growth are TLR1 and TLR6; however, their involvement can be minimized, since both require TLR2 to form heterodimers for their activity. Therefore, it seems unlikely that the phenotypes described here for MyD88 are mediated by TLR action. Based on the finding that IL-1β loss led to increased *S. aureus* biofilm growth in a previous report [13], [14], IL-1RI activity is likely involved since this receptor requires MyD88 for signaling. However, other MyD88-dependent receptors, such as IL-18R and IL-33R may also contribute to host immunity during biofilm development, which remains to be examined in future studies.

Here we demonstrate that biofilm-infected catheters become surrounded by an extensive host-derived fibrotic capsule rich in type I collagen and fibronectin. This endogenous host response to biofilms is often observed during human device-related infections [3], [22], [23], [24] and may impart additional survival advantages for biofilm growth and persistence. Indeed, this possibility is supported by our findings that the heightened fibrotic response in MyD88 KO mice was associated with increased *S. aureus* bacterial burdens and dissemination to distant organs during acute infection. In particular, the fibrotic capsule may provide a protective barrier to physically sequester bacteria from host immune recognition. Alternatively, fibrosis may facilitate *S. aureus* translocation and dissemination when organisms detach from the biofilm by virtue of their ability to produce adhesion molecules that demonstrate affinity for host fibrotic molecules as well as enzymes that degrade components of the ECM, basal membrane, and host tissues [25], [26]. Of note, recent evidence indicates that plasma or serum coating of artificial surfaces influences bacterial adherence [27]. Similarly, we have observed that catheter coating with host proteins enhanced *S. aureus* binding *in vitro* compared to non-coated catheters, with similar effects afforded by the ECM components fibronectin, hyaluronic acid, and heparan sulfate (Hanke and Kielian, manuscript in preparation). Therefore, it is likely that enhanced ECM deposition in MyD88 KO mice may facilitate *S. aureus* biofilm formation by providing additional substrate for bacterial colonization and/or dissemination. The dichotomy between the timing of elevated type I collagen and arginase-1 expression (i.e. days 7 vs. 14, respectively) in MyD88 KO mice remains to be resolved. Although significant increases in type I collagen were only detected at day 7 in MyD88 KO animals, it is likely that other ECM components such as fibronectin, hyaluronic acid, heparan sulfate proteoglycans are altered at both intervals to account for the exaggerated fibrotic response observed in MyD88 KO mice. It is important to note that arginase-1 is also expressed in fibroblasts in addition to M2 macrophages; therefore, the contribution of fibroblast arginase-1 remains to be determined in this system. Nonetheless, our results have established that MyD88-dependent signals play an important role in dictating the extent of fibrosis and M2 macrophage polarization during *S. aureus* biofilm development.

The host immune response plays a critical role during both physiological (i.e. wound healing) and pathological (i.e. hepatic and pulmonary) fibrosis by releasing several pro-fibrotic cytokines and other molecules that participate in ECM remodeling [17], [28], [29]. Fibrosis has been linked to the transition of macrophages into an alternatively activated M2 phenotype [17], [28], which is a key contributor during fibrotic reactions [30]. Interestingly, we observed a correlation between exaggerated fibrosis in MyD88 KO mice and enhanced M2 macrophage accumulation. This enhanced polarization of MyD88 KO macrophages to a M2 phenotype was also confirmed *in vitro* after exposing cells to *S. aureus* biofilms. Recent studies suggest that certain pathogens favor the transition of the immune response from a proinflammatory to an anti-inflammatory state [31], [32]. Although we previously reported that *S. aureus* biofilms are associated with a M2 macrophage infiltrate, the current study extends these observations to reveal a contribution for MyD88 in preventing excessive M2 macrophage recruitment and fibrosis. Because M2 infiltrates were not enhanced in TLR2 or TLR9 KO mice and the remaining TLRs do not recognize *S. aureus* motifs, this indicates that MyD88 acts independently of TLR pathways, which is another novel finding of the current study. Interestingly, a prior report demonstrated increased lung fibrosis following *C. pneumoniae* infection in IL-1RI KO mice, which also utilizes MyD88 for signaling, although the frequency of M2 macrophages was not examined [33]. In agreement with our findings, another recent study has revealed a role for IL-1β in regulating *S. aureus* burdens in a mouse model of orthopedic infection [14], presumably via actions emanating from the IL-1RI, although again, any effects on macrophage infiltrates or fibrosis were not investigated. Intriguingly, we do not observe much evidence of neutrophil influx into *S. aureus* biofilms using this infection model for reasons that remain unclear [34].

Another interesting finding in this study was that MyD88 loss had no effect on cytokine/chemokine expression during early stages of biofilm infection. This suggests that MyD88-independent mechanisms are responsible for inflammatory mediator production during acute biofilm infection; however, the molecule(s) that drive this early response remain to be elucidated. In contrast, inflammatory mediator release was significantly attenuated in MyD88 KO animals during later stages of infection, which was not observed with TLR2 or TLR9 KO mice in prior studies [13], [14]. Reductions in cytokine/chemokine expression during infection can often result from lower bacterial burdens in tissues. However, the decreases in IL-6, IFN-γ, and CXCL1 levels in MyD88 KO animals could not be explained by bacterial burdens, since *S. aureus* titers were either equivalent (catheter-associated) or higher (surrounding tissues) in MyD88 KO compared to WT mice at day 14 (Fig. 1). This suggests that MyD88-dependent mechanisms are responsible for regulating cytokine/chemokine levels during late infection, the identity of which remains to be defined. This is yet another example of the divergence of TLR and MyD88 action in the context of *S. aureus* biofilm infection. It is also interesting to note that MyD88 is not as critical for host immunity against *Staphylococcus epidermidis* (*S. epidermidis*) biofilm infections (data not shown). Specifically, our studies have revealed only a transient increase in *S. epidermidis* burdens associated with infected catheters and surrounding tissues of MyD88 KO mice at day 3 post-infection, which return to levels equivalent in WT animals by day 7. This contrasts with our findings for *S. aureus*, where titers remained significantly elevated at day 14 post-infection in tissues of MyD88 KO mice. In addition, there was little evidence of *S. epidermidis* colonization of the heart or kidney from the primary site of biofilm infection, whereas significant bacterial dissemination was detected following *S. aureus* biofilm infection in MyD88 KO animals at days 3 and 7. This discrepancy between MyD88 involvement during *S. aureus* vs. *S. epidermidis* biofilm infections may be explained by the fact that the latter is less pathogenic than *S. aureus*.

Collectively, these findings suggest that the loss of MyD88-dependent signaling exaggerates M2 macrophage polarization and fibrosis during *S. aureus* biofilm formation, which culminates in enhanced bacterial growth and dissemination during acute infection. By extension, this suggests that approaches designed to augment MyD88-dependent signaling may be efficacious for the treatment of patients suffering from persistent biofilm infections. Taken in the context of previous studies with TLR2 and TLR9 KO animals where these indices were not affected, the current report advances our understanding of MyD88-dependent cascades in dictating inflammatory mediator release that do not involve TLRs.

## Materials and Methods

### Ethics Statement

This study was conducted in strict accordance with the recommendations in the Guide for the Care and Use of Laboratory Animals of the National Institutes of Health. The protocol was approved by the Institutional Animal Care and Use Committee of the University of Nebraska Medical Center (Approval ID: 09-049-00-EP). All surgery was performed under avertin anesthesia, and every effort was made to minimize suffering.

### Animals

MyD88 KO mice (originally from Dr. S. Akira, Osaka University, Suita, Osaka, Japan) were obtained from the Centre de La Recherche Scientifique and have been previously backcrossed with C57BL/6 mice for over 10 generations [35], [36], [37]. Mice were housed in a restricted-access room equipped with ventilated polypropylene microisolator cages and maintained at 21°C under a 12 h light:12 h dark cycle with ad libitum access to water (Hydropac™; Lab Products, Seaford, DE) and Teklad rodent chow (Harlan, Indianapolis, IN).

### Mouse Model of *S. aureus* Catheter-associated Biofilm Infection

*S. aureus* biofilm infections were performed as previously described using a USA300 LAC strain chromosomally transduced with the luciferase gene *lux* (USA300 LAC::*lux*) [13], [38], [39]. Briefly, a small subcutaneous (s.c.) incision was made in the left flank under anesthesia and a blunt probe was used to create a pocket for insertion of a sterile, 14-gauge teflon intravenous catheter, 1 cm in length (Exel International, St Petersburg, FL). The incision was sealed using Vetbond Tissue Adhesive (3M, St. Paul, MN) and 10^3^ cfu USA300 LAC:*lux* in 20 µl of sterile PBS was slowly injected through the skin directly into the catheter lumen. The health status of the mice was regularly monitored throughout the course of infection and any moribund animals were immediately euthanized.

### Quantitation of *S. aureus* Burdens on Infected Catheters and Surrounding Tissues

At days 3, 7, 14, and 21 post-infection, MyD88 KO and WT mice were sacrificed by an overdose of inhaled isoflurane and catheters were removed using aseptic technique and placed in 1 ml of PBS for sonication to dissociate bacteria from the catheter surface. Tissues surrounding infected catheters were also collected, weighed, and disrupted in 500 µl homogenization buffer [PBS supplemented with 100 µl RNasin and a protease inhibitor tablet (Roche Diagnostics, Indianapolis, IN) using a Tissuemizer (Kinematica AG, Littau/Lucerne, Switzerland). To evaluate the impact of MyD88 on biofilm dissemination, *S. aureus* burdens were also quantitated in the kidney and heart following tissue disruption using a Bullet Blender (Next Advance Inc., Averill Park, NY). Bacterial titers associated with catheters and surrounding tissues, as well as systemic organs, were enumerated using TSA plates supplemented with 5% sheep blood (Hemostat Laboratories, Dixon, CA) and were expressed as Log_10_ cfu/ml for catheters or Log_10_ cfu/g wet tissue weight.

### Immunofluorescence Staining and Microscopy

Tissues surrounding infected catheters were fixed in 10% formalin and embedded in paraffin, whereupon 10 µm thick sections were deparaffinzed in xylene and a graded series of alcohols followed by antigen retrieval as previously described [13]. Sections were processed for immunofluorescence staining using primary antibodies specific for type I collagen (Millipore, Billerica, MA), fibronectin (US Biological, Swampscott, MA), and arginase-1 (Santa Cruz Biotechnology, San Diego, CA), followed by donkey anti-rabbit-FITC or -biotinylated secondary antibodies (Jackson ImmunoResearch Laboratories, West Grove, PA), and a streptavidin-594 conjugate for the latter (Invitrogen, Carlsbad, CA). Confocal imaging was performed using a Zeiss 510 META laser scanning microscope (Carl Zeiss, Oberkochen, Germany) with staining specificity confirmed by incubating tissues with a primary isotype-matched control antibody and appropriate secondary antibody. Importantly, isotype control stained tissues consistently revealed no background signals, indicating that *S. aureus* protein A was not influencing our antibody staining profiles. H&E images of catheter-associated tissues were captured using a Ventana Coreo Au slide scanner (Ventana Medical Systems Inc., Basel, Switzerland). The width of the host fibrotic response, as well as quantitation of arginase-1 and type 1 collagen fluorescence staining surrounding biofilm-infected catheters in MyD88 KO and WT mice was calculated from at least 12 random fields of view using AxioVision software 4.8 (Carl Zeiss, Oberkochen, Germany).

### Multi-analyte Microbead Array

To compare the expression of inflammatory mediators associated with biofilm-infected tissues of MyD88 KO and WT mice, a custom-designed mouse microbead array was utilized according to the manufacturer’s instructions (MILLIPLEX; Millipore, Billerica, MA), which detects the following inflammatory mediators: IL-1α, IL-1β, TNF-α, IFN-γ, IL-6, IL-9, IL-10, IL-12p40, IL-12p70, IL-15, IL-17, CXCL1, CXCL2, CXCL9, CXCL10, CCL2, CCL3, CCL4, and CCL5. Results were analyzed using a Bio-Plex workstation (Bio-Rad, Hercules, CA) and normalized to the amount of total protein recovered to correct for differences in tissue sampling size.

### Flow Cytometry

Tissues surrounding biofilm-infected catheters were collected and processed for flow cytometry as previously described [13]. Cells were stained with the following Abs: F4/80-PE-Cy7, Ly6G-AF700, and CD206-APC (all from BD Biosciences). iNOS (Abcam, Cambridge, MA), IRF-5 (Novus Biologicals, Littleton, CO) and arginase-1 (Santa Cruz Biotechnology) were detected by intracellular staining using either anti-goat-FITC or anti-rabbit-PE secondary Abs (Santa Cruz Biotechnology). Results are reported as the absolute number of F4/80^+^ macrophages after normalization to adjust for the recovery of different cell numbers from MyD88 KO and WT mice. Subsequently, macrophages were characterized as alternatively activated M2 cells on the basis of CD206 and arginase-1 expression, whereas classically activated M1 macrophages were identified as iNOS and IRF-5 positive [40], [41], [42].

### Macrophage-*S. aureus* Biofilm Co-cultures and qRT-PCR

USA300 LAC-GFP static biofilms and bone marrow-derived macrophages (BMDM) from MyD88 KO and WT mice were prepared as previously described [43], [44]. A total of 10^7^ MyD88 KO or WT BMDM were co-cultured with *S. aureus* biofilms at 37°C for 2 h under static aerobic conditions and based on enumeration of bacterial densities within the biofilm, this equated to a MOI of 10∶1 (bacteria/macrophage). Macrophage-biofilm co-cultures were harvested by mechanical dissociation after the 2 h co-culture period, and subsequently incubated with a F4/80 antibody and the live/dead stain 7-aminoactinomycin D (7-ADD; eBioscience, San Diego, CA), whereupon viable macrophages (F4/80^+^, 7-AAD^−^) were collected by FACS. MyD88 KO and WT BMDM exposed to the same manipulations but without bacterial treatment were included as a control. Total RNA was isolated from FACS-purified macrophages using a TaqMan Pre-Amp Cells-to-Ct kit (Applied Biosystems, San Diego, CA) and qRT-PCR performed for iNOS, TREM-1, TREM-2, IL-1β, IL-4, IL-10, CD206, arginase-1, TGF-β, type I collagen and fibronectin using ABI Assays on Demand primer/probe sets. Results were normalized against the housekeeping gene GAPDH and are presented as the fold-change (2^−ΔΔCt^) relative to unstimulated macrophages as a reference standard.

### Statistical Analysis

Significant differences between experimental groups were determined using the Student’s *t*-test with Welch’s correction for unequal variances (GraphPad Prism 4.03, GraphPad Software, Inc., La Jolla, CA). For all analyses, a *p*-value of less than 0.05 was considered statistically significant.
